# The Phytochemical Composition and Molecular Mechanisms Involved in the Wound Healing Attributes of *Bulbine* Species—A Critical Review

**DOI:** 10.3390/plants14193045

**Published:** 2025-10-01

**Authors:** Mxolisi P. Voko, Abdulazeez A. Ogbe, Manoj G. Kulkarni, Roger M. Coopoosamy, Johannes Van Staden

**Affiliations:** 1 Department of Nature Conservation, Faculty of Natural Science, Mangosuthu University of Technology, P.O. Box 12363, Jacobs, Durban 4026, South Africa; vokomxolisi@gmail.com (M.P.V.); rogercoopoosamy@gmail.com (R.M.C.); 2 Research Centre for Plant Growth and Development, School of Life Sciences, University of KwaZulu-Natal Pietermaritzburg, Private Bag X01, Scottsville 3209, South Africa; dammy2324@yahoo.com (A.A.O.); kulkarnim1@ukzn.ac.za (M.G.K.)

**Keywords:** *Bulbine frutescens*, *Bulbine natalensis*, *Aloe*, flavonoids, anthraquinones, knipholones, wound healing

## Abstract

*Bulbine* species (Asphodelaceae) are routinely used in many African communities to treat various dermatological disorders, including wounds, due to their relative accessibility, affordability, safety records, and reported efficacies. However, these reported biological activities lack robust empirical evidence and well-validated cellular mechanisms for plausible applications. Hence, this review was aimed at investigating the bioactive compounds of *Bulbine* species linked to their cellular wound healing attributes, their toxicity, and cytotoxicity. A detailed literature search was conducted using Web of Science, Google scholar, and PubMed, followed by Scopus and VOSviewer (version 1.6.20) bibliographic analyses. *Bulbine frutescens* (L.) Willd. and *Bulbine natalensis* Baker safely mediate tissue healing and coagulation cascade as adaptogens and cytotoxic agents. The wound healing activities of the *Bulbine* species were linked to the synergistic wound healing or tissue repair properties of bioactive compounds (such as saponins, terpenoids, luteolin, and apigenin) via the expression of collagen type-I, alpha-2 (*COL1A2*) gene, collagen III, increase in the wound tensile strength, and anti-cytokine interleukin-10 (IL-10) mRNA. *Bulbine* species were also reported to contain specialised biomarker compounds (such as naphthoquinones, bulbine-emodin, and aloe-emodin) which mediate the activation of hydroxyproline, Aryl Hydrocarbon Receptor, transforming growth factor beta—β1 (TGFβ1), and the suppressor of mothers against decapentaplegic proteins (SMAD), which ultimately induce tissue granulation, myofibroblast differentiation, re-epithelialization, higher protein complexes, and scar tissue formations. These findings give credence to the wound healing therapeutic potential of *Bulbine* species. However, additional clinical studies are necessary to further ascertain the reported efficacies of *Bulbine* species’ bioactive principles, their overall safety, and the underlying cellular mechanisms involved in the wound healing process and carcinogenesis.

## 1. Introduction

Africa is a tropical continent; thus, it serves as a hub for more than 55 common skin diseases [1]. Among these conditions are fungal infections (e.g., candidiasis, mycids, and tinea corporis); bacterial infections (e.g., leprosy, secondary syphilis, impetigo, and folliculitis); eczema (e.g., infectious pityriasis alba); viral infections (e.g., warts, herpes, and HIV); miscellaneous skin diseases (e.g., urticaria, acne, psoriasis, and malignant melanoma); autoimmune disease (e.g., alopecia areata and vitiligo); and parasitic infections (e.g., leishmaniasis and scabies) [1,2]. Most deaths due to skin-related diseases are reported in sub-Saharan African nations (e.g., Lesotho, Swaziland, Mozambique, South Africa, and Botswana). According to the 2025 world rankings, Lesotho (5.11%) holds the highest mortality rate caused by skin diseases in Africa after Grenada (12.60%) (https://www.worldlifeexpectancy.com/). In 2017, South Africa witnessed 1025 (i.e., 0.2%) deaths due to skin and subcutaneous tissue disorders [3]. Despite comparatively low mortality rates, skin ailments like wounds and injuries are increasingly becoming a major health concern due to their multifaceted nature. They affect the quality of life of all age groups, present exorbitant everyday medical burdens, increase governmental expenditures and allow the invasion of communicable diseases, opportunistic infections, and HIV/AIDS [1,4,5].

As Western medication remains unaffordable to many people [6], it is estimated that 80% of the world’s population relies on medicinal plants for basic healthcare [7]. According to the comprehensive list of African medicinal plants, Africa is also home to more than 5400 plant taxa with over 16,300 medicinal uses [8]. This implies that 10.8% of this African flora is used for traditional medicinal purposes, and 2942 species administered to humans originate from southern Africa [8]. The demand for medicinal plants and their products is high in South Africa. Recently, it was reported that over 200,000 tonnes of medicinal plant products are traded annually, with a turnover of about US$60 million [8]. It has also been reported that 70% of indigenous people consult traditional healers for primary healthcare, which depends on the scarce supply of medicinal plants [9]. Chemical antioxidant analogues such as butylated hydroxytoluene, butylated hydroxyanisole, and nonsteroidal inflammatory drugs like aspirin and diclofenac sodium are readily commercialised and currently being used [10]. However, sometimes these drugs have unbearable side effects [10,11]. Furthermore, finding cost-effective, less sophisticated, and readily available treatment options can be challenging for most resource-poor villagers. Thus, globally, indigenous people deem medicinal plants the best treatment alternative for various skin disorders and underlying comorbidities.

**Figure 1:**
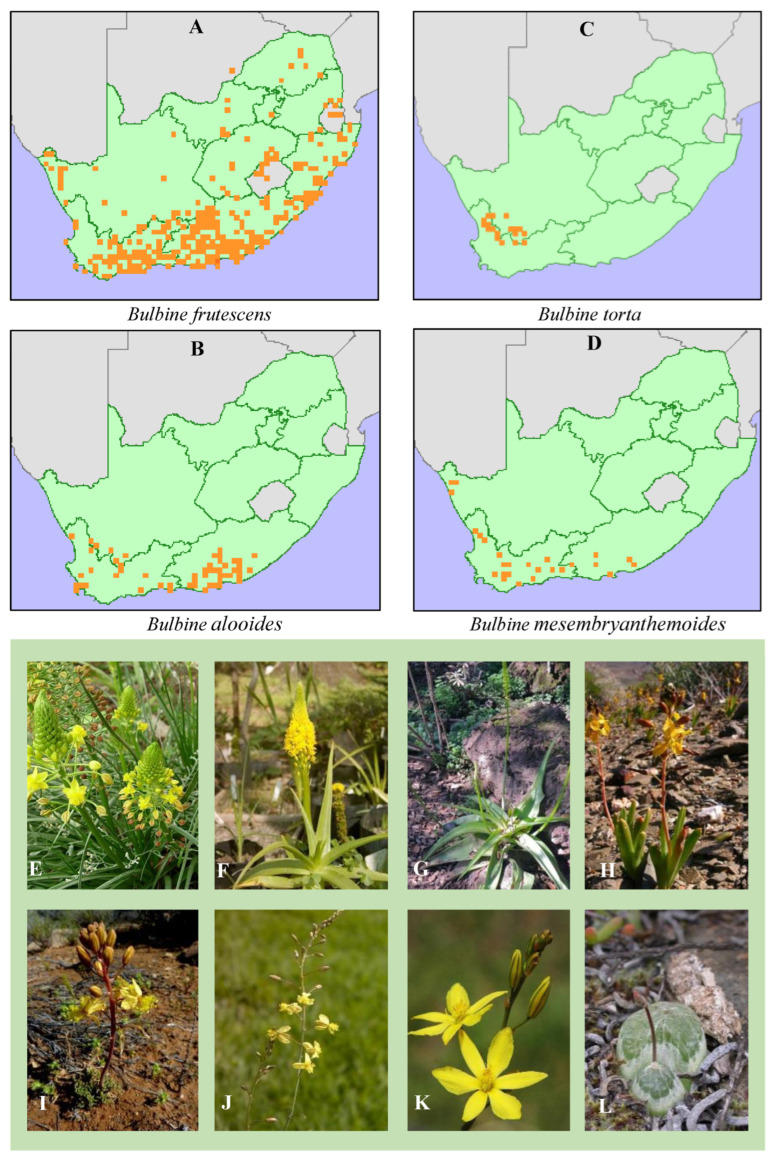
Recorded distribution of medicinal *Bulbine frutescens*, *Bulbine alooides*, *Bulbine torta*, and *Bulbine mesembryanthemoides* in South Africa with validated (**A**,**B**) and/or not (**C**,**D**) biological activity related to skin diseases and wound healing. The inserted orange colour on the map (**A**–**D**) represent each Bulbine species below the map. Letters (**E**–**G**) represent the most- and (**H**–**L**) least-studied *Bulbine* species in the genus *Bulbine* of the family Asphodelaceae: (**E**) *Bulbine frutescens* (L.) Willd., (**F**) *Bulbine natalensis* Baker (syn.: *Bulbine latifolia*), (**G**) *Bulbine alooides* (L.) Willd., (**H**) *Bulbine succulenta* Compton (**I**) *Bulbine torta* N.E.Br., (**J**) *Bulbine favosa* (Thunb.) Schult. & Schult.f, (**K**) *Bulbine bulbosa* (R.Br.) Haw., (**L**) *Bulbine mesembryanthoides* Haw. subsp. *Mesembryanthoides* (https://redlist.sanbi.org/; https://www.pacificbulbsociety.org/ and https://www.agaveville.org/) (Accessed 20 December 2023).

Among the most valued medicinal plants in African traditions, especially in South Africa and its neighbouring countries, are the *Bulbine* species, used for managing various skin conditions (Figure 1). The genus *Bulbine* of the family Asphodelaceae is endemic to the southern (67 species) and tropical (5 species) regions of Africa [12]. Forty *Bulbine* species are found in South Africa [11]. These include *Bulbine narcissifolia* Salm-Dyck, *Bulbine alooides* (L.) Willd., *B. frutescens* (L.) Willd., and *B. natalensis* Baker, which are now used around the world to prepare different ethnobotanical recipes [13]. Six *Bulbine* plants also grow in parts of Australia and New Zealand (Figure 1) [13]. *Bulbine* species share the Asphodelaceae family with the genus *Aloe* [14]. A study undertaken by Coopoosamy [6] showed that *Bulbine* and *Aloe* species contain similar chemotaxonomic biomarkers, responsible for enhancing skin health and wound healing. Interestingly, it is shown that traditional herbalists and healers, or the so-called ‘iNyanga’ or ‘iSangoma’ in South Africa, prepare various decoctions, infusions, or tinctures from *Bulbine* species as natural remedies for herpes, cracked lips, rashes, ringworm, burns, and wounds [15]. Though most wound healing claims of *Bulbine* species carry ethnobotanical significance in traditional medicine, they seem rather superficial and often lack ethnopharmacological explanations and scientific depth in the literature for plausible evidence of their dermatological relevance. For example, most studies overlooked the effect of *Bulbine* species on tumour necrosis factor α (TNF-α), interleukin-1 (IL-1) from leukocytes, and nuclear factor kappa B (NF_κ_B), responsible for the modulation of inflammation inducers or enhancers [16,17]. Yet abnormal skin functions of inflammatory cells (e.g., macrophages, basophils, neutrophils, and eosinophils) are pharmacologically attenuated by bioactive constituents of some medicinal plants. Establishing a scientifically based therapeutic value of *Bulbine* species is indispensable for rigorous quality control, cost-effective drug discovery, and the identification of specialised phytochemicals as antigenic compounds for skin ailments and wounds. Despite being poorly studied at the cellular level, *Bulbine* species have proven to be biologically active for wound healing [6,18,19]; hence, the aim of this review was to investigate the phytochemistry of *Bulbine* species linked to cellular wound healing attributes using chemotaxonomic signature markers and VOSviewer for comparative bibliographic analyses. We also reviewed the antimicrobial activities, cytotoxicity, and toxicity of *Bulbine* plants on carcinogenic and non-carcinogenic cell lines under in vitro and in vivo settings.

## 2. Methodology

One hundred and forty peer-reviewed articles were used to carry out bibliometric analysis and elucidation of mechanisms underlying *Bulbine* species ethnopharmacology (Supplementary Figure S1). Investigation into the ethnobotanical uses, phytochemistry (e.g., chemotaxonomic signature markers, flavonoids, quinones, and their pathways), histopathological evidence, antimicrobial properties, and possible wound healing mechanisms of action of *Bulbine* species were considered after removing duplicate and irrelevant reports. The cosmeceutical uses of *Bulbine* species in the management of dermal inflammation, promotion of hemostasis, tissue proliferation, and tissue remodelling, as well as their toxicological records, were also examined to understand the complex gene regulation involved in wound healing mechanisms. A search of the published literature using keywords such as “Asphodelaceae, *Bulbine* species, *Aloe*, ethnomedicinal uses, antimicrobial activity, cytotoxicity, inflammation, wound healing, and biomarkers” was undertaken. Reputable databases (e.g. Google Scholar, Scopus, PubMed, Web of Science, and ScienceDirect) were carefully searched for peer-reviewed articles, book chapters, dissertations, and official reports (Supplementary Figure S1). The coverage of the analytical search included the important literature published from the 1970s to 2025. An empirical-based method by Reddy et al. [20] was followed, using data from Scopus. Thereafter, GraphPad Prism (version 10.6.0) was used to visualise spatial data by country and subject area. VOSviewer (version 1.6.20) was used to carry out bibliographic analysis by subject, region, ethnobotanical use, assay, and thematic maps based on the citation overlay, authorship, and co-occurrence of text in the titles and abstracts. As a powerful bibliographic analytical tool, VOSviewer generates patterns of the data in the form of networks describing author contributions, countries, and terms reported in the literature for important associations [20]. VOSviewer was configured to exclude irrelevant phrases and repetitions of phrases or titles from the analysis.

## 3. Bibliographic Results and Discussion

**Figure 2:**
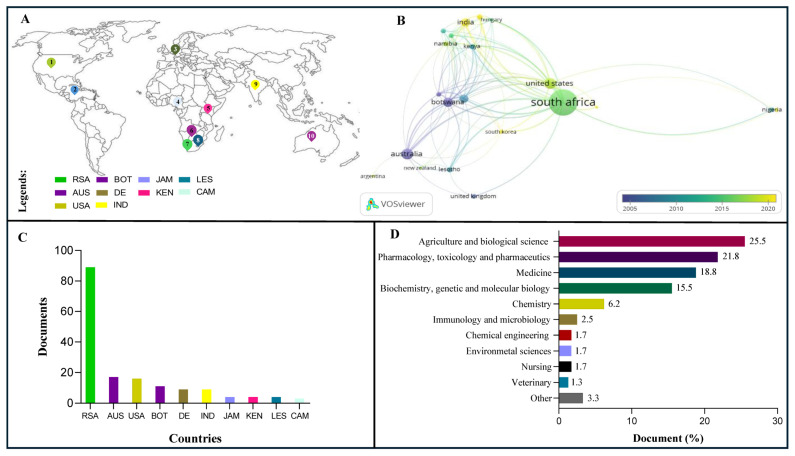
Spatial bibliographic analyses of *Bulbine* species studied in relation to wound healing and management of skin ailments. (**A**) Top ten countries conducting *Bulbine* research on skin ailments and wound healing. (**B**) Overlay visualisation of the most prolific research network in *Bulbine* research based on country affiliation (from 0 to 363-citation network). (**C**) Contribution of countries by publications (1–89 articles). (**D**) Contribution of authors by subject area (1–64 publications). Countries: RSA—Republic of South Africa; AUS—Australia; USA—United States of America; BOT—Botswana; DE—Germany; IND—India; JAM—Jamaica; KEN—Kenya; LES—Lesotho; CAM—Cameroon. The data were curated by document relevance from Scopus peer-reviewed articles (*n* = 140). Network: bubbles connected by lines denote association. A larger bubble indicates greater frequency of co-occurrence and importance. Thicker lines connecting two bubbles indicate a greater number of links for the item in the network.

Whilst the origins of *Bulbine* and *Aloe* remain debatable, the two genera are popular in African phytomedicine [12]. Spatial bibliographic analysis of *Bulbine* species reveals that research trends relating to wound healing and management of skin ailments are found in 31 countries (i.e., 15.90% of all 195 nations) (Figure 2A). An overlay visualisation network of Scopus data showed that South Africa is the most engaged and leading country, publishing 89 articles between the 1900s and 2025 (Figure 2B,C). This is evident by the larger margin of reports originating in the region and higher collaboration indexing in VOSviewer analysis. For example, bibliographic analysis of peer-reviewed articles (*n* = 140) showed that authors from South Africa have strong affiliation links with researchers from Australia, the United States, Botswana, and Germany, which occupy the top five countries, with 17, 16, 11, and 9 publications, respectively. This link may have contributed to the increased number of reports in the United States, Germany, Australia, and India and may be used to promote more research engagement. In contrast, Vietnam, Sweden, New Zealand, the Republic of China, and Egypt showed less engagement, with one article between the 1900s and 2025. The publications of Van Wyk [8], Motsei et al. [21], and Thring and Weitz [22] from the *Journal of Ethnopharmacology* and *South African Journal of Botany* were the top cited papers, with 363, 360, and 268 citations, respectively (https://scholar.google.com/). These findings suggest that Asphodelaceae species reported in these papers can be viewed as important for top research areas such as agriculture, biological science, pharmacology, toxicology and pharmaceutics, medicine, biochemistry, molecular biology, and phytochemistry (Figure 2D). These were research areas where individual authors contributed an average of 1–64 articles.

**Figure 3:**
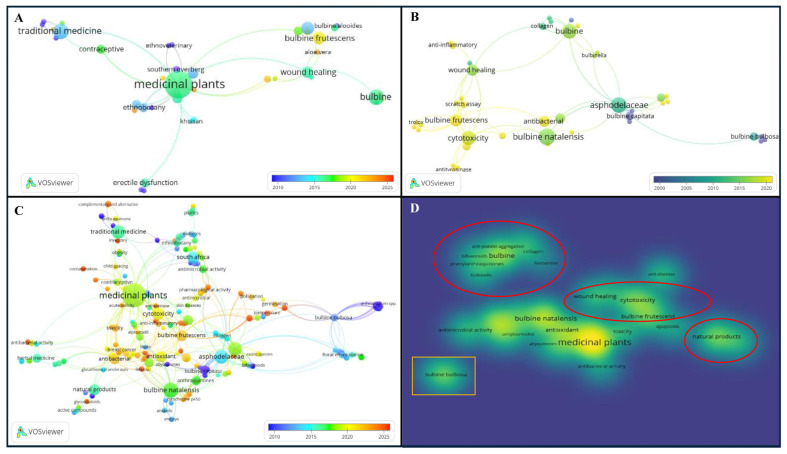
Summary of bibliographic analysis of the literature on *Bulbine* species used for wound healing and management of skin disorders from the late 1900s to 2025. (**A**) Network visualisation of ethnomedicinal uses of *Bulbine* species for managing skin ailments. (**B**) Feature-based overlay visualisation network map of wound healing bioassays conducted to validate *Bulbine* ethnomedicinal uses. (**C**) Term map of research related to *Bulbine* with network visualisation of the trends in citations over time. (**D**) Density thematic map of wound healing based on the co-occurrence of terms in both the title and abstract fields using 140 publications based on *Bulbine* research.

**Figure 4:**
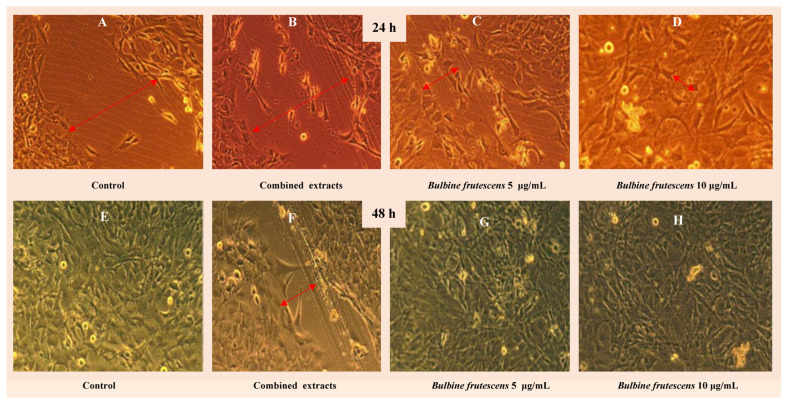
Wound healing and closure 24-and 48 h after exposure to *Bulbine frutescens* (L.) Willd. extracts. After 12 h (**A**–**D**), a larger degree of wound closure was obtained from *B. frutescens* at all concentrations (**G**–**H**) compared to the control (**A**,**E**). Wound closure after 48 h was observed in all samples exposed to *B. frutescens* and the control (**E**), except the combined extracts (**F**). The figure was generated using information and images from Keele [25].

Given the scope of this review, it was evident that the top three most cited articles partly elucidated the role of *Bulbine* species in promoting healthy skin and wound healing, but sparsely discussed the mode of action of the bioactive compounds. Additionally, Motsei et al. [21] explored the antifungal activities of *B. frutescens* (L.) Willd and *B. natalensis* Baker against *Candida albicans*, while Thring and Weitz [22] surveyed *B. lagopus* (Thunb.) N.E.Br. (syn.: *B. asphodeloides* (L.) Spreng.) ethnomedicinal uses in Bredasdorp/Elim (South Africa). Thring and Weitz [22] further established that both *A. ferox* and *B. lagopus* are common treatments for several skin disorders, including eczema, wounds, irritations, burns, herpes, bruises, and venereal sores [22]. Since *A. ferox* and *Bulbine* species have some inherently similar chemical constituents, the healing activity of *Aloe* species (e.g., *A. vera*, *A. arborescens*, and *A. marlothii*) is comparable to the therapeutic effect elicited by *Bulbine* plants, especially the synergistic healing properties of their leaf gels. The similarities in the species’ phytochemical signatures not only suggest the parallel biochemical activity of the two genera but also justify most of our bibliographic outcomes and findings from different bioassays (Figure 3 and Figure 4).

The wound healing properties of *Bulbine* plants have been attributed to the polysaccharides and glycoproteins components of their leaf gel, as well as to their hydrating properties [23], thus giving credence to the patented *B. frutescens* gel (12.5–25%) for commercialisation. Additionally, pantheol and asiaticoside are also known to enhance scar tissue formation [24]. The results of our feature-based over-visualised network map (based on citation over time and term co-occurrence in titles and abstracts) suggest that *B. frutescens* and *B. natalensis* were the most reported species investigated for their antimicrobial, antioxidant, anti-inflammatory, and cytotoxicity properties in a bid to scientifically validate their wound healing and skin disease management capacities. Furthermore, the reported wound healing activity of *B. natalensis*, *B. frutescens*, and *B. capitata* Poelln. can be associated with the presence of anthraquinones (e.g., phenylanthraquinones), abyquinones, and cytochrome P450 regulation (Figure 3C,D). Our density thematic map showed four important clusters related to the species phytochemistry and different biological activities. These included (i) *Bulbine* species inducing wound healing through platelet aggregation and collagen production; (ii) the association of *B. frutescens* with wound healing, cytotoxicity, and apoptosis; (ii) investigation endeavours aimed at drug discovering natural products; and (v) the association of *B. natalensis* mostly with anti-plasmodial, antioxidant, and anti-bacterial activity (Figure 3).

### 3.1. Ethnobotany of Bulbine Species

Table 1 highlights the various ethnomedicinal uses of the most studied *Bulbine* species (i.e., *B*. *frutescens*, *B. asphodeloides*, and *B. natalensis*) in southern Africa, especially in South Africa. This followed spatial bibliographic analysis of *Bulbine* species by studies showing strong country network maps between African countries (e.g., South Africa, Botswana, Lesotho, Kenya, and Nigeria) (Figure 2A–C). It is for these reasons that we chose to further discuss the traditional uses, preparation, and administration methods of *B. frutescens*, *B. asphodeloides*, and *B. natalensis* in southern Africa. Although empirical evidence relating to the traditional dermatological uses of Bulbine species is well-documented, it primarily focuses on wound healing (Table 1). Our bibliographic analyses revealed that people of African descent primarily use *Bulbine* plants to treat wounds, sunburns, scars, rashes, and other skin conditions. However, after the 19th century, their ethnomedicinal uses broadened among the rural communities to cover mouth ulcers, diarrhoea, stomach ailments, and other conditions [6]. It is therefore not surprising that, similar to *B. frutescens*, *B. natalensis* Baker (syn.: *Bulbine latifolia* (L.f.) Spreng.) is traditionally used to treat different skin diseases (Table 1). It attracted a lot of attention through its ability to treat multiple skin conditions, such as eczema and ringworm [26,27]. Interestingly, ethnobotanical studies revealed that aside from the antirheumatic records of *B. natalensis*, traditional healers in South Africa also administer *B. natalensis* to HIV/AIDS-positive patients as adaptogens and against opportunistic antifungal skin infections [26].

#### 3.1.1. Wound Healing Properties of *Bulbine* Species

Wounds are classified as open or closed depending on the underlying cause, and also as acute or chronic wounds depending on the healing anatomical physiology [34]. Open wounds are sub-classified into incised wounds, tear wounds, and abrasions or superficial wounds, avulsions, puncture wounds, and penetration wounds [35,36]. Owing to the nature of open wounds and cracked skin, the skin is often more susceptible to bleeding and infections, which can lead to prolonged or delayed healing [34]. *Bulbine* species promote healing in different wounds, bruises, or cuts through numerous mechanisms involving the repairing of muscles, bruises, blood vessels, and via the inhibition of pathogenic organisms that delay healing (Table 2). For example, leaf gels from *B. frutescens* and *B. natalensis* are traditionally used to stop bleeding and induce the adaptogenic healing of burns, itches, cuts, mosquito bites, ringworm, and wounds (Table 1). The reported traditional uses of *B. frutescens* leaf gel include healing of acne, rashes, insect bites, cold sores, mouth ulcers, cracked lips, and blisters [37,38]. The use of leaf gel as an anti-clotting agent is noteworthy, as this is a crucial mechanism in wound healing and tissue repair. According to Coopoosamy [6] and Van Wyk [32], the Xhosa people in the Eastern Cape also use the roots and bulbs of the two species as treatment agents against scrofula. It is worth noting that indigenous people from South Africa use 15% of medicinal plant taxa from Asphodelaceae as natural remedies for skin disorders [39]. This makes medicinal plants of the Asphodelaceae family the most valuable (next to the Asteraceae family at 23%).

#### 3.1.2. Traditional Methods of Preparing *Bulbine* Species for Wound Healing Purposes

Although there is limited data on the effective dosage and administration methods for *Bulbine* species used in managing various dermatological conditions, our findings agree with the findings of Otang et al. [26] who revealed that the bark (15%), roots (21%), and leaves (43%) were the most commonly used plant parts in the Eastern Cape for treating dermatological conditions and diseases. Our results further suggest that leaves and roots are used in varying forms (Table 1). This is in accordance with the findings of Otang et al. [28] who reported that *Bulbine* species can be prepared in various forms, such as decoctions, infusions, and lotions, when used to manage skin diseases. These forms are either prepared using leaves or roots from species such as *B. frutescens*, *B. natalensis*, *B. narcissifolia*, and *B. asphodeloides* with greater distribution in South Africa (Figure 1; Table 1). Similarly, oral and topical applications were reported as the most popular methods of administration, followed by bathing and enemas. For instance, the leaves of *B*. *natalensis* are applied as poultices to treat burns, itches, rashes, and wounds, while leaf sap or gel is first warmed before application onto wounds, burns, itches, and rashes [39].

## 4. Phytochemistry and Pharmacological Activities of *Bulbine* Plants

The findings of Kibiti and Afolayan [11] revealed that *Bulbine* species like *Bulbine abyssinica* A.Rich. contain free radical quenching potential and protein degradation inhibitory agents, which confer a higher protection against oxidative damage caused by reactive oxygen species (ROS) and erythrocyte membrane lysis. This is attributed to their analytes having high levels of phytochemical constituents such as anti-nutrients, phenols, alkaloids, flavonoids, flavanols, and proanthocyanidins [11].

### 4.1. Anti-Nutrients

*Bulbine* species like *B. abyssinica* have phytate, oxalate, and vitamins (A, C, and E). Though phytic acid and oxalates are considered anti-nutrients because of their conjugation property into insoluble complexes with proteins and minerals, they possess great medicinal value [44]. For example, phytate can intercept the inflammation process, inhibit platelet aggregation, and is a known suppressant of insulin resistance [45]. This property can be useful in the attenuation of non-healing wounds and ulcers of diabetic patients. Oxalates (oxalic acid), on the other hand, are deemed detrimental to the human body when consumed in excess owing to their strong binding affinity with Na^+^, Ca^2+^, and Mg^2+^ [46]. This often leads to a cation deficiency required for normal physiological functioning of the human body [46]. Nonetheless, to allay these fears, researchers have noted that the oxalate content of *Bulbine* species is generally low, and the plants are usually used externally after undergoing a heating process. Remarkably, oxalates are heat-labile; thus, during traditional extract or sap preparations, additional oxalates are lost to heat [39].

### 4.2. Tannins and Saponins

Phenolics are the most ubiquitous group of non-enzymatic antioxidants found in high concentrations in medicinal plants. Their *innate* biological functions include anti-ageing, anti-inflammation, anti-carcinogen, anti-atherosclerosis, and wound healing [15]. Most phenolics are amino acid derivatives with an aromatic ring that has one or more hydroxyl groups, which form negatively charged phenolate ions. It is via these phenolate ions that hydrogen and ionic bonds can be formed with human proteins and DNA [47]. The interaction between phenolate ions and human cellular components enables pharmacological bioactivities, including anti-inflammatory and anti-microbial activity. Similarly, saponins and tannins have become popular anti-inflammatory, anti-microbial, anti-mutagenic, anti-hemolytic, and anti-diabetic agents [15]. Tannins, for example, possess more than 10 hydroxyl groups and can be divided into (i) non-hydrolysable tannins (with more hydroxyl groups) formed by catechin polymerization or dimerization and (ii) hydrolysable tannins with two subgroups (namely, gallotannins and ellagitannins) (reviewed by Prinsloo et al. [47]). Metabolomics analysis of *B. abyssinica* revealed significant relative abundances of saponins and tannins [11]. *Bulbine abyssinica* contains terpenoids (e.g., carvone), mainly known for their antioxidant activity in wound healing through radical chelation mechanisms [19,48]. Similarly, metabolite profiling of *B*. *natalensis* led to the identification of tetracyclic triterpene, *β*-sitosterol, and two pentacyclic triterpenes, namely glutinol and taraxerol [49]. These phenolic compounds are well known to induce tissue repair in superficial skin, burn, and/or wound healing assays [9,25,37,38]. However, investigations have shown that tannins can hinder physiological processes by precipitating functional enzymes due to their non-specific enzymatic nature [9,48]. Nevertheless, the moderate protein-binding affinity and antioxidant activities of many tannin-rich Asphodelaceae members give credence to the enhanced wound healing activities recorded in the species [9]. Furthermore, the increased wound healing attributes of *Bulbine* species could be associated with their saponin contents [9], known to intrinsically orchestrate anti-inflammatory action via the inhibition of cyclooxygenase-2 (COX-2), tissue necrosis factor, pro-inflammatory cytokines (e.g., Interleukins 1 and 6), and synthesis of prostaglandins [50].

### 4.3. Flavonoids

**Figure 5:**
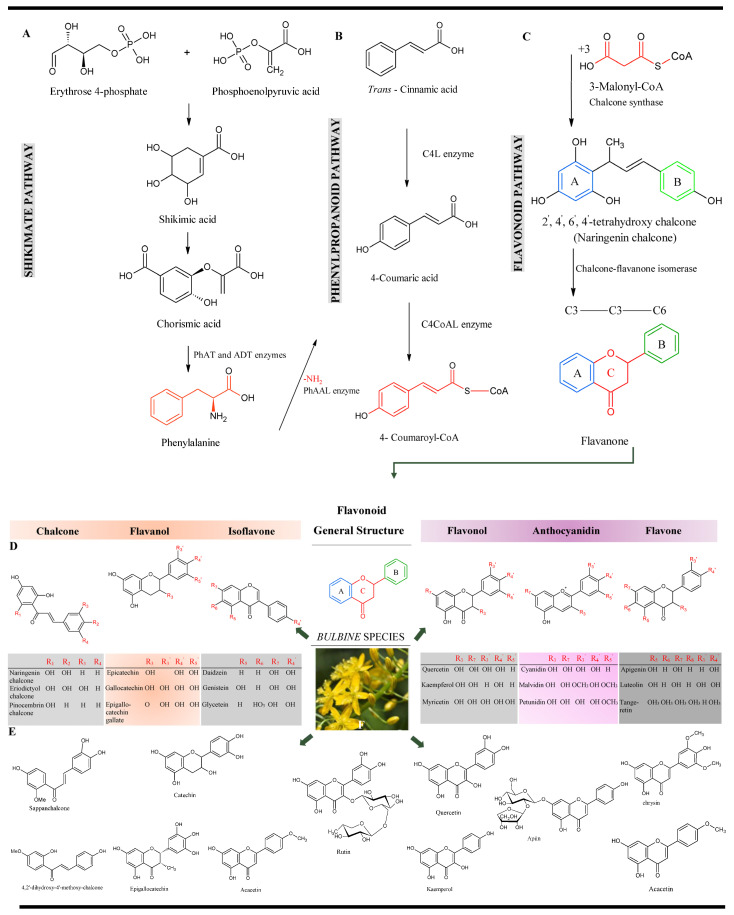
Putative flavonoid pathways in medicinal plants: (**A**) shikimate pathway, (**B**) phenylpropanoid pathway, and (**C**) flavonoid pathway. (**D**) Examples of flavonoids. (**E**) Presence of some of the flavonoids in *Bulbine* species, with or contributing to anti-inflammatory and injury healing activity, and (**F**) *Bulbine natalensis* Baker. Initially, the acetate pathway (generating ring A) and the shikimic pathway (generating ring B) merge to form a heterocyclic pyran ring C, yielding the C6-C3-C6 flavonoid skeleton [51,52]. This originates with the aldol condensation reaction of phosphoenolpyruvic acid and D-erythrose 4-phosphate [53]. Chorismic acid is converted into phenylalanine by prephenate-aminotransferase (PhAT) and arogenate-dehydratase (ADT) enzymes [54,55]. The phenylpropanoid pathway initiates from phenylalanine in the presence of phenylalanine ammonia-lyase (PhAAL) which deaminates phenylalanine to form *trans*-cinnamic acid [52,54,55]. The cinnamate-4-hydroxylase (C4L) hydroxylates *trans*-cinnamic acid into 4-coumaric acid (p-coumaric acid), from which the enzymatic action of 4-coumarate-(C4CoAL) mediates the biosynthesis of 4-coumaroyl-CoA to produce coumarin skeleton [54]. A step-by-step condensation of 4-coumaroyl-CoA under mediation by chalcone-synthase activity, ultimately produces 2′, 4′, 6′, 4-tetrahydroxy chalcones [53,55]. Chalcone isomerase converts chalcone to flavanone/naringenin from which flavonoid subclasses are then synthesised [53].

Flavonoids are the most active subgroup of phenolics in their activation of different wound healing mechanisms (Figure 5).

This observation was recently supported by Cedillo-Cortezano et al. [56] and Mssillou et al. [57]. Six important subclasses of flavonoids, namely chalcones, flavanols, isoflavones, flavonols, anthocyanidins, and flavones, have been reported in *Bulbine* species (Figure 5D,E). Though flavonoids are defined as simple structures (Figure 5A–C), each compound varies in substituent at different C—positions formed by hydroxylation (larger OH groups), methylation (more common methoxy groups in ring B compared to ring A), acylation, and glycosylation with monosaccharides or oligosaccharides. The common hydroxylation pattern in flavonoids is found at C-5 and C-7 in ring A, and C-4′ in ring B, which usually makes a functional group catechol when there is an additional hydroxyl group at C-3 (ring B) [54]. It is via these variant C positions of functional groups and backbone structures that flavonoids enable the induction of unique chemical and physical properties such as phytoalexins, photoreceptors, allelopathy, antioxidant, and antimicrobial activities [58,59]. In addition to quenching ROS, orthodihydroxy ring-B-substituted flavonoids inhibit free radicals by chelating metal ions and as reducing agents of xanthine oxidase, responsible for generating superoxide anion [60]. Flavonoid–metal complexes also imitate superoxide dismutase activity [60].

### 4.4. Specialised Biomarker Compounds

**Figure 6:**
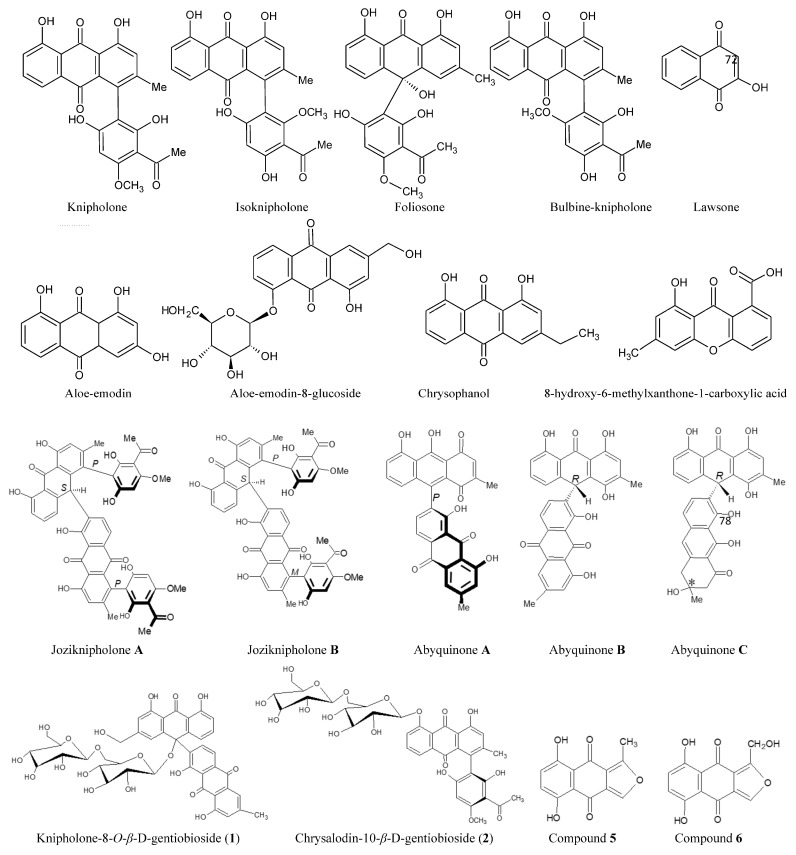
Specialised biomarker compounds reported in *Bulbine* species. Readers are encouraged to refer to the original reports cited in this review for more information on the putative chemical structures. Chemical structures were generated using the data of Bringman et al. [66], Qhotsokoane-Lusunzi and Karuso [65], Wanjohi et al. [67], and Bezabih et al. [68].

The genus *Bulbine* from the Asphodelaceae family possesses unique chemotaxonomic markers distributed between seven to nine genera [61,62]. Of these compounds, l,8-dihydroxyanthraquinones are ubiquitous in *Bulbine*, *Bulbinella*, and *Kniphofia* [62]. Following the identification of knipholone and isoknipholone, the three genera were considered monophyletic (Figure 6). The antioxidant power and pharmacological properties of species from the Asphodelaceae family appear to depend on the monomeric anthraquinones (such as laccaic acid, chrysophanol, aloe-emodin, islandicin, and aloe-emodin acetate); dimeric anthraquinones (such as asphodelin, knipholone, chryslandicin, and rare dimeric phenylanthraqunones joziknipholones A and B); oxanthrones (such as isofoliosone and foliosone); and phenyl anthraquinones and anthrones (such as knipholone anthrone, isoknipholone anthrone, knipholone, phenylanthrone knipholone anthrone, and anthraquinone isoknipholone) [63].

#### Anthrones and Anthraquinones

Van Staden and Drewes [64] reported the first biaryl anthraquinone knipholone **1** isolate from fresh bulbs of *B. natalensis* and *B. frutescens*. Acetosyringone, chrysophanol, knipholone, isoknipholone, 10,7′-bichrysophanol, and chrysalodin and anthraquinone glycosides [knipholone-8-*O*-*β*-D-gentiobioside (**1**) and chrysalodin-10-*β*-D-gentiobioside (**2**)] were later isolated in *Bulbine narcissifolia* [65]. However, nuclear magnetic resonance spectroscopic elucidation showed that the chemical structure of compound **1** binds weakly to DNA [65], suggesting lower antioxidant activity.

Meanwhile, the efficacy of the isolated bulbine-knipholone (**6**), abyquinone C, abyquinone B, (10*R*)-1,4,8,1′,8′-pentahydroxy-3,3′-dimethyl-[10,7′-bianthracene]-9,9′,10′(10H)-trione and abyquinone A (*P*)-8,9,1′,8′-tetrahydroxy-3,3′-dimethyl [10,7′-bianthracene]-1,4,9′,10′-tetraone justified the use of *B. abyssinica* as a treatment for body rashes, eczema, fungal infections, and venereal diseases [66,67]. Contrary to the findings of Qhotsokoane-Lusunzi and Karuso [65], *B. natalensis* methanolic extract (with pentacyclic triterpenes, tetracyclic triterpene, *β*-sitosterol, pheophytin A, and knipholone) showed better reducing power at 250 µg/g [49]. The FRAP assay may have allowed these signature compounds to elicit a higher ferric-reducing power of Fe^3+^ to Fe^2+^. This can be corroborated by the colorimetric property of phenolics and other polar components in the methanolic extract [49]. The structural configuration of these compounds provides a logical explanation for their bioactivity. For instance, the attachment of a hydroxyl group at C-3 next to the double bond at C-5 and C-6, for example, in sterols and triterpenes, enhances the antioxidant activities of the compounds [69].

Furthermore, 4′-Demethylknipholone, isoknipholone, knipholone, chrysophanol, foliosone, 5,8-dihydroxy-1-hydroxymethylnaphtho [1,3-c]furan4,9-dione, luteolin, and apigenin were reported in *B. capitata* [70]. 4′-demethylknipholone (C_12_H_16_O_7_) is a dark red solid compound with 420.0835 mass and exhibits maxima at wavelengths of 202, 224, 254, 287, and 430 nm in the UV-Vis spectrum [70]. Using ^1^H NMR for structural resonance, Bezabhi and Abegaz [70] identified 4′-demethylknipholone as a biogenetic precursor of knipholone (compound **2**) and isoknipholone (compound **3**). This was ascribed to ^1^H NMR of 4′-demethylknipholone lacking methyl resonance and a 14 amu lesser MS compared to putative knipholone [70]. Phenyl anthraquinones were reported in *B. capitata* [68,71] and *B. frutescens* [72] as a class of anti-plasmodial and anti-cancer compounds [49,73]. Interestingly, *B. capitata* is traditionally used in Botswana as an antibiotic and antipyretic agent, which is thought to derive its phytotherapy from root isofuranonaphthoquinones belonging to not more than 18 compounds [68].

## 5. Wound Healing Mechanisms of *Bulbine* Compounds

As far as we know, not all wound healing bioactive compounds of *Bulbine* species have been isolated. However, using chemotaxonomic signature markers, histopathological evidence, and immunohistochemistry findings, we can decipher active compounds that may be responsible for wound healing in *Bulbine* species. These natural compounds promote wound healing and anti-inflammation by improving the stimulation of cell proliferation, differentiation of keratinocytes, and dermal fibroblasts and through the induction of nitric oxide synthase expression and collagen synthesis (e.g., alpha-type 1 type 1 collagen) [57,74]. Controlling oxidation and inflammation caused by increased free radicals that damage wound recovery proteins is an essential step of the wound healing process. The presence of the inducible isoform cyclooxygenase-2 (COX-2) increases in response to wound growth factors and cytokines. These pro-inflammatory molecules can impose similar consequences on wound healing as free radicals [75,76]. Cyclooxygenase-2 expression, biofilm pathogens, and free radicals delay the wound healing process. Therefore, the anti-inflammatory, anti-microbial, and antioxidant activities exerted by medicinal plants form a scientific basis for their wound healing capabilities [77,78,79]. It is for this reason that plant-derived phenolics are the most reported phytochemicals in wound healing studies [57]. For example, previous reports from wound healing and protein precipitation assays show that higher protein binding affinity occurs when phenolics such as tannins permeate the interfibrillar peptide chain at many positions, resulting in the formation of a film that later develops into a physical barrier, which is vital in wound healing processes [9].

### 5.1. Wound Healing Activity of Flavonoids

The most common mechanisms by which flavonoids exert their wound healing properties are perhaps through their antioxidant and anti-inflammatory activities. Flavonoids chelate with radicals and cause adhesion as well as the augmentation of fibroblasts, thereby having an approbatory effect for cell recovery [25,57]. Flavonoids, including rutin, quercetin, apigenin, luteolin, and chrysin, have all been identified from *Bulbine* species as wound healing supportive compounds. Similarly, flavonoids including lucenin 2, orientin, isovitexin, and vicenin 2 were reported by researchers as the main components of many *Aloe* species, and they have been implicated in accelerating wound healing processes. It has been suggested that these compounds, as found with *Bulbine* species’ wound healing-related bioactive compounds, enhance wound healing via angiogenesis, re-epithelialization, antioxidant, and anti-inflammatory mechanisms [80,81].

#### 5.1.1. Quercetin and Rutin

Flavonols are an important subgroup of flavonoids, with ketone groups and building blocks of proanthocyanins. Compared to flavones, flavonols have a hydroxyl group in position 3 of the C-ring that can be glycosylated [82]. The complex nature of specialised central metabolism results in flavonols having diverse bioactivities due to methylation and hydroxylation [82]. For example, having the OH-group at the C-3 position of the flavonoid chemical structure may explain why flavonols display more efficacy in chelation and inhibiting ROS accumulation [83]. This also results in rutin and quercetin in particular inducing better anti-injury and anti-inflammatory actions via the inhibition of monoamine oxidases (MOA) and reduction in gamma-aminobutyric acid (GABA) levels [50]. The presence of acetyl and rhamnosyl groups in flavonoids like isorhamnetin, with quercetin derivatives, brings about additional anti-acetylcholinesterase activity [50]. This activity increases acetylcholine, which is responsible for tissue repair, reduced scarring, angiogenesis, and faster re-epithelialization [50]. Thus, it can be argued that flavonoids of *Bulbine* species are involved in different chemical signalling pathways that mediate wound healing mechanisms in damaged mammalian tissues.

Flavones and flavonoids also encode for other signalling pathways, which also elicit wound healing effects via the upregulation of interleukins (e.g., mainly IL-10), M2 macrophage, CD31, and vascular endothelial growth factor-α (VEGF-α), as well as downregulatory mediation of inflammatory mediator interferon-β INF-β, matrix metalloproteinases (MMP-2 and MMP-9), and tumour necrosis factor-α (TNF-α) [57]. For instance, a study by Özay et al. [84] reported that kaempferol ointments accelerated wound recovery in diabetic mice by enhancing hydroxyproline and collagen synthesis, accelerating re-epithelialization and wound closure. Similarly, compelling findings were obtained with quercetin, icariin, and morin [57]. Quercetin, quercetin 3-*O*-(6-malonyl-glucoside) 7-*O*-glucoside, kaempferol-3-*O*-rutinoside, rutin, variabiloside A, eriodictyol, astilbin, pratensein, ᴪ-yectorigenin, petunidin, petunidin-3-*O*-rutinoside, and yanidin-3-*O*-rutinoside flavonoids were among the suite of key bioactive compounds detected in *Bulbine* species like *B. abyssinica* leaves (Figure 5D–F) [19]. Therefore, based on the known activities of these flavonoids, they are likely to be responsible for orchestrating different wound healing activities [19,85].

#### 5.1.2. Luteolin and Chrysin

Flavones are found as glycosides in the fruits, leaves, and flowers of plants [82]. Despite being variably ubiquitous [48], only the aerial parts of *B. capitata* showed apigenin and luteolin (Figure 5) [81]. Luteolin from *B. capitata*, *Rosmarinus officinalis*, and *Origanum vulgare* showed high wound healing activities [70,86]. Furthermore, luteolin in medicinal plants was reviewed by Mssillou et al. [57] activated wound healing by downregulating cytokines, mainly inflammatory mediators, interleukins (IL1ß, IL-2, IL-6, IL-8, IL-12, and IL-17), INF-β, and TNF-α, and selectively upregulates mRNA expression of anti-inflammatory cytokine IL-10. An in vivo study showed that luteolin reverses delayed wound healing by targeting cellular and molecular pathways which elicit skin wound healing by accelerating re-epithelialization in diabetic rats [87]. In addition to its anti-oxidative and anti-inflammatory properties, luteolin, at the cellular level, increases ubiquitin carboxy-terminal hydrolase (UCH)-L1 expression and stops the cascade expression of vascular endothelial growth factor [87]. Topical application of luteolin analogue in streptozotocin-induced mice prompted rapid wound healing via re-epithelialization and lowered the mediation of Nrf2 oxidative response and NF-κB-inducible inflammatory response. Flavone chrysin also imposed positive effects on wound healing and inflammation by inducing the expression of genes responsible for healing by P53, MMPS, and IL-6 [88]. Investigations by Almeida et al. [89] and Pivec et al. [90] also showed that rutin activates similar wound healing activity as luteolin in its ability to reduce oxidative stress after a trauma or injury.

#### 5.1.3. Apigenin

Among glycosaminoglycans (GAGs) and elastin, which constitute the dermal matrix of adult skin, type I (80–85%) and type III collagen (10–15%) are the major components [91]. Alpha-1 type-1 is encoded by *Col1α*(I) gene. This gene promotes wound healing by coding for the stimulation of the pro-alpha-1 (I) chain, a component of type 1 collagen [74]. The pro-alpha-1 (I) chain integrates with another pro-alpha-1 (I) chain as well as the pro-alpha-2 (I) chain to synthesise the type I pro-collagen molecule, which then undergoes complex cellular processes and rearrangement to produce type 1 collagen fibres [74]. Zhang et al. [91] showed that apigenin (4,5,7-trihydroxyflavone) from medicinal plants induced wound healing by promoting the expression of mRNA of collagen [type I, alpha 2 (*COL1A2*)] and collagen [type III, alpha 1 (*COL3A1*)] in NIH/3T3 and human dermal fibroblast (HDF) cells. It was also apparent that apigenin rapidly expressed phosphorylated suppressor of mothers against decapentaplegic 2 (*SMAD2*) and *SMAD3* proteins in a dose-dependent fashion, activating the *SMAD2*/*3* signalling pathways. This resulted in an increased collagen synthesis and, ultimately, dermis recovery. Further examination of apigenin and luteolin on human HaCaT cells also showed protective effects against UV irradiation damage [92]. The degradation of collagen under the cellular matrix metalloproteinases (MMPs) mediation occurs in UV irradiation damage to the skin. This study showed that apigenin and luteolin alleviated the irradiation-induced damage and UV-inducible collagenolytic MMP-1 expression by suppressing UV-induced phosphorylation of Ca^2+^/calmodulin-dependent protein kinases (CaMKI and CaMKII), which are upstream modulators of MAPK pathways [93]. The two compounds also lowered the expression of c-Jun, c-Fos, and activation of activator protein 1 (AP-1) signalling in cultured HaCaT cells [93]. This led to the conclusion that apigenin and luteolin can serve as biological compounds for wound healing and reconstructive skin rejuvenation agents [91]. Though these findings are appealing, further research is still required to elucidate the molecular mechanisms of wound healing by apigenin, rutin, and luteolin from *Bulbine* in vitro and in vivo.

### 5.2. Wound Healing Properties of Bulbine Signature Compounds

One of the compelling findings of wound healing properties in *Bulbine* species is from the quinones. Aloe-emodin, shikonin, alkannin, and lawsone (2-hydroxy-1,4-naphthoquinone) act similarly to epigallocatechin-3-gallate and ellagic acid as ROS quenchers, lipid peroxidation suppressants, and antioxidant enzyme [superoxide dismutase (SOD), glutathione (GSH), catalase (CAT) superoxide dismutase (SOD), glutathione (GSH), and catalase (CAT)] stimulants [92].

#### 5.2.1. Effects of *Bulbine* Signature Compounds on Transforming Growth Factor-β

The ability of quinones to regulate transforming growth factors-β (TGF-β) and *SMAD proteins* for successful wound healing is an interesting element in understanding the role plant secondary metabolites play in wound healing. Transforming growth factors-β are fibroblast mitogenic [37]. Unlike the TGF-β2, which occurs earlier and TGF-β3—expressed in later stages of healing—TGF-β1 is the most abundantly expressed isoform by degranulating platelets at the wound site to provide early signals for tissue repair [94]. TGF-β1 codes for a major and multifaceted reprogramming of epithelial and mesenchymal cells to promote the expression and extracellular accrual of matrix proteins that elicit rapid restoration of tissue integrity after the injury or disruption of tissue barriers. By acting on fibroblasts, TGF-β 1 triggers the transcription of various matrix proteins, including fibronectin and triple helical protein ‘collagens’ [94]. The transcription then mediates cellular migration to wound target sites and initiates stimulation of cytokines/chemokines responsible for granulation tissue formation [95]. Emodin from *R. officinale* and aloe-emodin influenced TGF-β1, which justified accelerated cell migration to the wound target site, resulting in new blood vessel formation, extracellular matrix synthesis, and better wound contraction on the skin after 24 h [94,96]. Interestingly, anthraquinone aloe-emodin was one of the signature biomarker compounds (knipholone, knipholone anthrone, islandin, and chrysophanol) identified in roots of *B. abyssinica* and *B. frutescens* [97]. The isolated emodin and aloe-emodin from these *Bulbine* species may have indirectly induced wound healing activities in a similar fashion (Figure 4C,D and Figure 6). Keele [25] examined the in vitro wound healing activity of *B. frutescens* using a scratch assay on 3t3-L1 cells and ascertained that 5 and 10 µg/mL of extract was responsible for faster cell migration and proliferation, which resulted in faster wound closure. Though the wound healing properties of *B. frutescens* suggest induction by polysaccharides and/or glycoproteins [38], these phytochemicals would have promoted wound healing via antioxidant activity and increased cell proliferation and migration of keratinocytes (Figure 4) [98]. However, the possibility of an epigenetic role of *Bulbine* anthraquinones cannot be overlooked.

#### 5.2.2. Effects of *Bulbine* Signature Compounds on SMAD Proteins

The study by Tang et al. [94] confirmed the effect of emodin on SMADs (SMAD2, 3, co-SMAD4, and SMAD7) as intracellular proteins phosphorylated by TGF-β receptors to transduce signals to specific TGF-β-inducible genes. SMAD proteins are signal transduction carriers of TGF-β-mediated mechanisms. Tang et al. [94] carried out a Western blot gene analysis and revealed that emodin simultaneously triggered *SMAD2* and *SMAD3* gene protein expression in a decoupling manner that discriminates the downregulation of the *SMAD7* gene. SMAD7 is an antagonistic phosphorylation protein, which, similarly to *SMAD3,* strongly binds to the activated TGF-β type 1 receptor [99]. It can disrupt wound healing signalling pathways by forming a stable association with protein receptor complexes. This causes molecular dephosphorylation that then restricts *SMAD3* expression, contributing to disruption in TGF-β-mediated signalling [94]. These findings suggested the involvement of quinones (such as aloe-emodin and emodin) in the induction of transcriptional changes via TGF-β-inducible genes responsible for cell growth and proliferation, angiogenesis, and extracellular matrix turnover [94]. However, it should be noted that the expression of the *SMAD7* gene in other gene transformation studies was linked to promoting wound healing [100,101]. Therefore, the *SMAD7* gene requires further exploration to fully decipher its role in wound healing.

#### 5.2.3. Effects of *Bulbine* Signature Compounds on Mitogen-Activated Protein Kinases

The mitogen-activated protein kinase (MAPK) family underlies important regulatory processes of inflammation, proliferation, oncogenesis, and migration. For example, Jun-terminus kinase (JNK) activity plays an instrumental role in the migration of fibroblasts, while extracellular signal regulatory kinases (*ERK1/2*) are involved in cell proliferation and are critical for promoting angiogenesis [96]. The *P38* protein kinase group, on the other hand, is responsible for apoptosis, cell differentiation, and inflammation via inducible cytokine cell migration [96]. Molecular analysis of the MAPK pathway involved in wound healing revealed a significant upregulation of JNK and *P38* after treatment with emodin and aloe-emodin. There was a high specific binding potential for MAPK gene expression, especially by aloe-emodin [96]. Furthermore, a higher molecular docking score also validated a dose-dependent inhibition of aloe-emodin compared to emodin in the manner 2.5 µM > 5 µM > 10 µM [96]. The observed JNK1 and *P38* gene regulation implied that aloe-emodin (an important signature marker in *Bulbine* chemotypes) was more potent compared to emodin’s interaction with the MAPK enzyme. Authors attributed this to the presence of a hydroxyl methyl group in aloe-emodin instead of the hydroxyl group in the C-3 position of emodin, as well as the absence of a methyl group in aloe-emodin on the C-6 position [96]. This seems a rather ‘trivial’ chemical difference in functional groups at C-3 and C-6 positions, which suggests a functional relevance in molecular mechanisms that promote wound healing. Additionally, this highlighted the importance of anthraquinone functional groups, especially in aloe-emodin and emodin, as profibrinolytic and healing agents that also promote hydroxyproline synthesis [96].

### 5.3. In Vitro and In Vivo Studies Related to Remodelling Phase and Histopathology

In vivo studies and histopathological analysis by Tang et al. [94] and Lin et al. [102] revealed more immediate cutaneous wound healing and burns repair on the skin of mice treated with emodin and aloe-emodin. The authors observed an increase in the epithelialization and a significant reduction in re-epithelialization time in wounds with flattened rete ridges. *Aloe* chemotype-based properties are comparable to those detected in in vitro and in vivo assays of *Bulbine* species for wound healing (Figure 4) [34,98]. Pather et al. [38] and Pather and Kramer [37] reached similar conclusions after treating cutaneous wounds of female pigs with *Bulbine* extracts. Furthermore, after a biopsy, they observed a significantly faster re-epithelialization rate (measured histologically by keratinocyte migration to form neo-epidermis) in wounds treated with *B. natalensis* and *B. frutescens* leaf gels compared to the untreated control animals. Nevertheless, the authors attributed their results to unidentified bioactive compounds that may be responsible for influencing myofibroblast activity, keratinocyte proliferation, and facilitating collagen deposition, which are significant factors contributing to the enhanced wound healing observed in *Bulbine*-based treatments. Additionally, while keratinocytes are increasingly being proven as key promoters of proliferation and remodelling phases through re-epithelialization, collagen re-synthesis, and scarring maturation [103], their motility is linked to the expression of surface integrin receptors that interact with the components of the extracellular matrix [104]. In wound sub-areas, keratinocytes and macrophages synthesise TGF-β1 as a major wound healing modulator that induces migration, heparin-binding epidermal growth factor, and transforming growth factor-α (TGF-α), which then facilitates the migration, differentiation, and proliferation of different keratinocytes [104,105]. The intense in vivo immunolocalization of TGFβ-R1 and TGFβ-R2 in the epidermis of pigs treated with *B. natalensis* and *B. frutescens* was an evident indication of increased TGF-β binding receptor activity and existing moiety [37].

Hydroxyproline is an important signature index of collagen synthesis, given that it is almost exclusively found in collagen [94]. Successful wound contraction entails active collagen deposition and maturation from initial wounding to scarring. Findings from *Bulbine* species investigations support the idea that some anthraquinones and biological entities promote hydroxyproline synthesis, which helps wounds gain tensile strength during tissue repair. The merits of *B. natalensis* and *B. frutescens* for increased hydroxyproline were confirmed by the increased tensile strength of porcine skin wounds [37,38] as well as faster wound closure in 3t3-L1 and Vero cells in in vitro assays (Figure 4) [25]. The increased collagen production coincided with biochemically estimated increased hydroxyproline and protein contents in *Bulbine*-treated groups. These findings were comparatively similar to the 10% faster healing activity induced by *A. vera* on diabetic wounds of Wistar mice [106]. At 10 days, *B*. *natalensis*- and *B. frutescens*-treated wound tissues showed earlier re-epithelialization (collagen maturation) and collagen deposition [37]. Furthermore, *B. frutescens* induced α-smooth muscle actin (α-SMA) expression in pigs as a later wound healing signal, indicating that both the accumulation of fibroblasts and their differentiation into myofibroblasts increased extracellular matrix and collagen fibre formation [37]. In another study, emodin significantly increased hydroxyproline but also inhibited TNF-α-induced expression of MMP-1, which is a central enzyme involved in the degradation of the extracellular matrix (Figure 6) [107].

## 6. Antimicrobial Properties of *Bulbine* Species

Table 2 summarises the minimal inhibitory concentration (MIC_50_) of *Bulbine* species with anti-bacterial and antifungal activity mostly on wound pathogens. In a study by Ghuman et al. [16], *Bulbine* analytes showing MIC values of <1 mg/mL were considered to have good anti-microbial activity. Interestingly, irrespective of the organic solvent polarity, MIC_50_ values of *B. frutescens* leaf extracts showed excellent inhibitory activity against Gram-positive and Gram-negative bacteria strains found in wounds (Table 2). Authors attributed the low MIC_50_ values of *B. frutescens* on *Klebsiella pneumoniae*, *Acinetobacter baumanii*, and *Staphylococcus aureus* as a good indication of their wound healing properties [6,16,41]. Likewise, Seleteng-Kose et al. [43] reached a similar conclusion after observing the effect of *B. narcissifolia* extract (MIC_50_ = 0.04 mg/mL) on *Neisseria gonorrhoeae*. Given the upsurge in the demand for *Bulbine* bioactive agents as the main active ingredients in locally produced wound healing lotions and cosmeceutical creams [38], the anti-bacterial properties of anthraquinones such as chrysophanol may hold explanations for these *Bulbine*-based biological activities (Figure 6). For example, recently discovered rhizome anthraquinone derivatives in *B. natalensis*, namely bulbnatalonosides A–E and bulbnatalone, were isolated and evaluated for their anti-microbial activities [108]. Only bulbnatalonoside A was an antigenic inhibitor against methicillin-resistant *Staphylococcus aureus* (MRSA) at an IC_50_ of 0.02 µM [108]. Yet no inhibitory activity against MRSA was observed by Weideman [41] after investigating the methanolic, aqueous, and acetonic leaf extracts of *B. frutescens* (Table 2). This suggests that the antimicrobial property against the MRSA strain in *Bulbine* species could be both subterranean-dependent and chemotype-specific.

Studies investigating the antifungal activity of *Bulbine* species are limited in number. This was first pointed out by Bodede and Prinsloo [48], who reviewed the antifungal activity in *B. frutescens* linked to candidiasis therapy and *B. natalensis* used in the treatment of opportunistic fungal infections found in HIV patients. A few reports have shown that the bulbs and leaves of *B. natalensis* and *B. narcissifolia* are antagonistic to *Candida albicans*, *C. tropicalis*, and *Trichophyton mentagrophytes* (Table 2). The phytochemical constituents of these *Bulbine* species were responsible for inducing MIC of 0.31 and 0.63–1 mg/mL against *T. mentagrophytes* and *C. albicans*, respectively [16,42]. These findings revealed that the *Bulbine* plants exhibit antifungal activities at remarkably low concentrations. Additionally, other studies have highlighted the microbial inhibition caused by *B. natalensis* secondary metabolites (including phytosterols, ergosterol, sitosterol, and stigmasterol) against *Aspergillus flavus*, *Fusarium verticilloides*, and *Penicillium digitatum* [109]. Despite showing greater activity than control, ergosterol was the most repressive compound against the three fungal strains [109]. Tannins (such as pyrogallol and catechin) disrupt the normal physiological activities of some pathogenic fungi due to their high toxicity to most microbes [110]. Flavonoids, on the other hand, may have orchestrated antifungal activity by inhibiting the synthesis of nucleic acids to perturb cytoplasmic membrane functions (reviewed by Rachuonyo et al. [110]).

## 7. Toxicology Evidence of Some Wound Healing *Bulbine* Species

The prolonged usage of medicinal plants, including species of *Bulbine,* in the management of various ailments often raises safety and toxicity concerns [111,112]. Western healthcare practitioners generally vacillate in their recommendations of medicinal plants and herbal products, citing concerns about public health. Thus, toxicological investigations of medicinal plants are invaluable to allay the fears of the public [113]. Interestingly, a few contrasting studies have been reported on the toxicity potentials of some wound healing *Bulbine* species and their phytoconstituents.

Table 3 presents the toxicity reports of some *Bulbine species* used in the management of skin diseases. Methanolic extracts of *B. natalensis*, *B. abyssinica*, *B. frutescens*, *B. asphodeloides*, and *B. narcissifolia* all displayed non-cytotoxic effects towards keratinocytes (HaCaT) at all tested concentrations [114]. Similarly, extracts of *B. abyssinica* and *B. frutescens* [115]*, B. abyssinica* [116], and *B. asphodeloides* [117] all exhibited non-cytotoxic effects in vitro. In contrast, a quinone isofuranonaphthoquinone isolated from *B. frutescens* showed remarkable in vitro cytotoxicity against Jurkat T cells [118]. Comparatively, knipholone anthrone was about 70–480-fold more cytotoxic to leukemic and melanocyte cancer cell lines than knipholone [119].

In animal studies, histological evidence showed that repetitive ingestion of 25, 50, and 100 mg/kg body weight in healthy male mice was not completely safe for the liver and kidneys on the 14th day (reviewed by Bodede et al. [48]). The use of *B. natalensis* extracts suggested that the toxicity in the liver is higher than that of the kidneys. The liver showed distortions in liver lobules, and there were proximal convoluted tubules in the kidneys [120]. Moreover, a dosage of 25 and 50 mg/kg body weight for pregnant female rats was safe during the organogenic period, while *B*. *natalensis* extracts at 100 mg/kg per body weight induced indifferent gender toxicity on reproductive functions [120].

**Table 3:** Toxicity of some *Bulbine* species used for the management of skin disorders and wounds in South Africa.

*Bulbine* Species	Plant Part (s)	Safe Dosage	Toxic Dosage	Toxicity	Key Finding	Reference
*Bulbine abyssinica A.Rich.*	Whole plant	LD_50_: 3120 µg/mL oil and 0.0625–1 mg/mL (fractions)	-	No toxic effect	All the plant fractions were non-toxic with LD_50_ values greater than 1 mg/mL.	[115,116]
	Leaves	100–500 µg/mL	-	No toxic effect	*B. abyssinica* stimulated the proliferation of these cells can accelerate wound healing.	[114]
*Bulbine frutescens* (L.) Wild.	Whole plant	62.5 and 125 mg/mL (aqueous extract)	62.5 and 125 mg/mL (ethanol extract)	Hepatotoxicity	*B. frutescens* extracts increased glucose utilisation in Chang liver cells, except for toxic ethanolic extracts at 62.5 and 125 mg/mL.	[121]
	Leaves	100–500 µg/mL	-	No toxic effect	*B. frutescens* stimulated the proliferation of these cells can accelerate wound healing.	[114]
	Stem	25 mg/kg of body weight	50 and 100 mg/kg of body weight	Hepatorenal toxicity	Reduced kidney and liver weight, albumin and alkaline phosphatase.	[120]
*Bulbine natalensis* (L.) Baker (syn.: *Bulbine latifolia* Roem. Et Schult.)	Stem	25 and 50 mg/kg of body weight	100 mg/kg of body weight	Gonadotoxicity	100 mg/kg body weight decreased progesterone and reproductive functions in male and female rats.	[122]
	Leaves	100–500 µg/mL	-	No toxic effect	*B.natalensis* stimulated the proliferation of these cells can accelerate wound healing.	[114]

**Figure 7:**
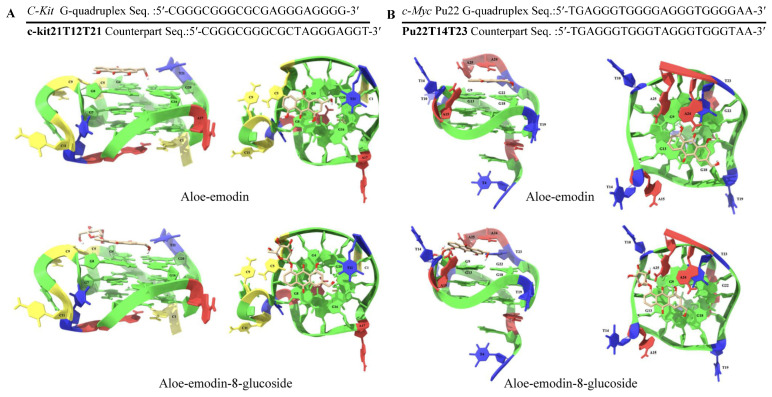
Putative binding mode of anthraquinone aloe-emodin and aloe-emodin-8-glucoside and complex formation with the (**A**) *C-Kit* G-quadruplex and (**B**) *c-Myc* G-quadruplex sequences [98]. Lateral and top views are presented by the (**left**,**right**) sides of (**A**), the same as in (**B**). Lateral (**left**) and top (**right**): views of the aloe emodin (**1**) and aloe emodin-8-glucoside. The nucleotides are visualised as slabs and filled sugars: red = adenine (A); yellow = cytosine (C); green = guanine (G); blue = thymine (T). Labels highlight key nucleotides, and only polar hydrogens are shown. The ligand molecules are rendered in sticks and coloured-coded in accordance with the atoms. The figure was generated using information from Dallalle et al. [98] and Das et al. [123].

*Bulbine* chemotaxomic markers showed anti-cancer activity beyond wound healing and through inherent interaction with G-quadruplex. This is a unique secondary nucleic acid structure with TTAGGG repeats in the telomere and genome [123]. It is understood as a canonical DNA conformation with four guanines held together by Hoogsteen hydrogen bonds, wherein individual guanine can act as both a donor and an acceptor for the two hydrogen bonds [124]. In vitro experiments showed the enrichment of G4 structures in genomic regions with a specific G4 motif (G_≥3_N_1-7_G_≥3_N_1-7_G_≥3_N_1-7_G_≥3_), three guanines per repeat or in a non-stringent G4 motif [124]. One of these loci is the oncogene promoter region *c-KIT* [125], *c-MYC* [126], *KRAS* [127] and *VEGF* [128]. Lately, it has become more apparent that targeting G-quadruplex (G4) with stabiliser molecules (i.e. G4 ligands) is a viable anti-cancer and wound healing strategy (Figure 7). This is because G4 ligands can modulate G4 structures’ formation or introduce genomic stability that shows anti-tumour activity by blocking the expression of oncogenes and cellular replication [124]. For instance, aloe-emodin and aloe-emodin-8-glucoside were reported as potent G4-binding molecules, especially on *c-KIT* and *c-MYT* enriched sequences, measuring in the 10^5^ order of binding affinity, which was greater than duplex DNA binding capacity (Figure 6 and Figure 7) [123]. These observations seem to cement the anti-carcinogenic activity of aloe-emodin and aloe-emodin-8-glucoside isolated from *Bulbine* species. This biochemical elucidation of ligand/nucleotide complex formation may offer a new way to design a drug from natural compounds with increased G4 stabilising activity [98].

## 8. Conclusions and Future Perspectives

In this study, our bibliographic analysis of *Bulbine* species revealed that research relating to the wound healing activities of these species is trending in South Africa, Australia, the United States, Botswana, and Germany. These plants are important to many human endeavours, including in agriculture, pharmacology, toxicology, pharmaceutics, medicine, biochemistry, molecular biology, and phytochemistry. Limited in vitro and in vivo studies have demonstrated that extracts of *B. abyssinica, B. frutescens*, and *B. natalensis*, as well as their purified active principles, possess therapeutic potential for wound healing and skin disease management. The wound healing mechanisms of action of *Bulbine* species’ bioactive compounds are highly complex at the cellular level. Although skin diseases and wound healing processes are generally considered complex and difficult to manage, *Bulbine* flavonoids and quinones have been shown to orchestrate wound healing by influencing the Aryl Hydrocarbon Receptor, TGF–β1, and SMAD proteins, thereby promoting tissue granulation, myofibroblast differentiation, re-epithelialization, and higher protein complexes. Other phytotherapeutic mechanisms are exerted via MOA inhibition, reduction in angiogenesis and GABA levels, as well as anti-inflammatory, antioxidant, and antimicrobial activities. *Bulbine* extracts and bioactive compounds can induce their pharmacological activities at acceptable safety levels while maintaining both antimicrobial activity and cytotoxic effect.

Novel wound dressing formulations offer several advantages over conventional dressings and have broader applications that can address some drawbacks of conventional dressings, such as solubility and limited activity at wound sites. Going forward, studies are needed to fully unravel the mechanism of action of the most promising *Bulbine* compounds, including flavonoids and quinones, while giving special attention to their effects on cell-surface receptors such as receptor tyrosine kinase. Emphasis in clinical trials should also be placed on wound healing *Bulbine* herbal formulations and their purified phytocompounds to confirm their efficacy and safety in the treatment management of skin diseases and conditions. Additionally, quality standardisation, biomarker validation, and the formulation of regulatory policies will be crucial for the effective integration of *Bulbine*-derived products into clinical practice for the benefit of patients.

## Figures and Tables

**Table 1:** Ethnomedicinal uses and traditional applications of *Bulbine* plant species in dermatology.

Bulbine Species	Common Name	Distribution	Ethnicity	Method of Preparation	Ethnobotanical Uses	Reference
*Bulbine abyssinica* A.Rich.	Geelkatstert, moetsa-mollo, ibhucu, and intelzi	Central and Eastern Africa from the Somali to Angola, Ethiopia, Eswatini, and South Africa.	Afrikaners, Zulus, Sotho, and Xhosa people	Tea and decoction	Decoctions from crushed roots are used against infertility problems and back pains, while ingesting leaves in tea form curbs coughs and bladder and vaginal infections. It is also used to manage diabetes and as livestock medicine (anti-helminthic for cattle).	[28,29]
*Bulbine asphodeloides* (L.) Spreng.	Copaiva, Balsamkopieva	KwaZulu-Natal, Lesotho, and the Eastern Cape	Zulus, Xhosas, Sothos and Mfengu people	Crush leaf to extract the gel	Leaf gel/juice is smeared on rough skin, sunburns, and also acts as an anti-ageing cosmetic. South Basotho people use leaf juice for dressing burns and cracked lips. Tubers are used to treat rashes, sores and wounds.	[17,28,30]
	Not reported	Eastern Cape, Free State and KwaZulu-Natal	Zulu and Xhosa people	Crush leaf to extract the gel	The leaf gel is also applied to fresh cuts, mosquito bites, and chapped lips, as well as on abrasions.	[31]
*Bulbine alooides* (L.) Willd.	Not reported	Eastern Cape and KwaZulu-Natal	Zulu and Xhosa people	Not specified	Tubers are used as treatments for urinary tract infections, rheumatism, syphilis, wounds, rashes, and diarrhoea, whereas roots treat venereal diseases.	[28]
*Bulbine frutescens* (L.) Wild.	Ibhucu	Eastern Cape	Xhosas, Khoisan, and Dutch settlers	Decoction and infusion	Roots and rhizomes are applied as scrofula treatment and extract as styptic.	[6,32]
	Intelezi, ingelwane, rankkopieva	KwaZulu-Natal and Limpopo	Zulus	Decoction and infusion	Treatment for fever blisters, cracked fingers, nails, heels, acne, insect bites, mouth ulcers, and genital sores. Also administered as cattle anti-helminthic.	[6,29]
	Snake flower, Geelkatstert	Not reported	Afrikaans	Not specified	Fresh leaf extracts are used as remedies for coughs, arthritis, insect bites, acne, colds, and for fast wound healing. Cosmeceutical relevance includes taken to treat burns.	[33]
	Ithethe elimpofu,	Free State, Northern Cape, and Western Cape	Zulus and Xhosas	Decoction and infusion	Roots are pulverised into decoctions as treatments for Xhosa children with convulsions. Zulus use root and leaf infusions as an emetic to mad patients.	[31]
	Rankkopieva	Lesotho	Sotho	Not specified	Leaves are used against sciatica and rheumatism.	[28]
*Bulbine narcissifolia* (L.) Salm-Dyck	Tloruthloru, khomo-ea basemane, kopiva	Lesotho, Gauteng, Free State, Western Cape, KwaZulu-Natal	Sotho, Griqua and Zulus	Infusion and decoction	Leaf sap applied as regimen for warts, ringworms, corns, and lip breakages. Cold infusion of leaf is administered as purgative whereas decoctions prepared from roots are administered to treat venereal diseases.	[28]
*Bulbine natalensis* (L.) Baker (syn.: *Bulbine latifolia* (L. f.) Roem. Et Schult.)	Ibhucu	Cape Floristic Region	Khoisan and Cape Dutch settlers	Not specified	Tubers are used as blood purifier and lumbago. Leaf sap is directly applied to treat eczema rashes, itches, wounds, and burns.	[6,17,18,32]
	Incelwani	Eastern Cape	Xhosas	Decoction	Leaf sap and roots are prepared into decoctions taken orally to treat diabetes, dysentery, eczema, rheumatism, and as an emetic.	[27]
		KwaZulu-Natal	Mfengu, Khoikhoi, Zulus, Shona and Xhosas	Decoction	Mfengu people used decoctions of dry root as remedies for lumbago, abdominal complaints, diarrhoea, dysentery, and syphilis. Leaf and leaf sap are used for septic wounds and eczema.	[28]
	Intelezi and ibhucu	Eastern Cape, KwaZulu-Natal, Zimbabwe, and Mozambique	Xhosas and Zulus	Infusion and decoction	Direct skin application of leaf sap treats wounds, burns, sores, rheumatism, ringworms, rashes, sunburns, and herpes.	[6,9]

**Table 2:** Minimal inhibitory concentration of *Bulbine* species extracts with anti-microbial activity.

Bulbine Species	Part Used	Extractant	Concentration (g/mL)	Anti-Microbial Activity (MIC_50_ mg/mL)			Reference
				Gram(+)Bacteria	Gram(-)Bacteria	Fungi	
*Bulbine asphodeloides* (L.) Spreng.	Inflorescence	Ethanol	2	Cm	Ec and 0.90 Xc	Not reported	[40]
	Roots	Ethanol	2	Cm	Ec and 0.90 Xc		
*Bulbine frutescens* (L.) Wild.	Leaves	Chloroform	0.05	0.63 Bs, Sp and Sa	0.63 Spy, Pv, Pa and Pm	0.63 Ca, Ct and Tm 1.25 Tr	[6,16,41]
	Bulbs	Chloroform	20	0.63 Se and Ab	0.63 Pm and 2.50 Ss		
	Leaves	Methanol	0.05	0.63 Sa	0.63 Pv and 2.50 Pa		
		Methanol	1.00	0.26 Sa and MRSA	0.26 Acb and 0.06 Klp		
	Leaves	Acetone	1.00	0.19 Sa, MRSA, 5.0 Bs and 2.0 Mk	0.06 Acb and Klp		
	Leaves	Aqueous solvent	1.00 and 0.50	0.43 Sa, MRSA and 2.0 Bs	0.43 Acb, 0.05 Klp, Esc and Pv		
	Roots	Aqueous solvent	0.50	3.0 Mk	Esc and Kla		
	Leaves	Acetone	0.05	2.0 Mk, 1.0 Bs and 1.0 Sa	Esc, Pv and Kla	Not reported	[6]
*Bulbine longifolia*		Ethyl acetate		5.0 Bs, 4.0 Mk and 3.0 Sa			
	Roots	Acetone	0.05	2.0 Bs, Mk and Sa	Esc, Pv and Kla		
		Ethyl acetate		7.0 Bs, 5.0 Mk and 5.0 Sa	Esc, Pv and Kla		
	Rhizomes	Acetone		1.0 Sa, 1.0 Bs and 2.0 Mk	Esc, Pv and Kla		
		Ethyl acetate		7.0 Bs, 6.0 Mk and 4.0 Sa	Esc, Pv and Kla		
	Leaves	Acetone	0.05	1.0 Bs, Mk and Sa	3.0 Esc, 3.0 Pv and Kla	Not reported	[6]
		Ethyl acetate		3.0 Bs, 2.0 Mk and 2.0 Sa	Esc, Pv and Kla		
*Bulbine natalensis* (L.) Baker	Roots	Acetone	0.05	2.0 Bs, Mk and Sa	Esc, Pv and Kla		
	Rhizomes	Acetone	0.05	2.0 Bs, 1.0 Mk and 1.0 Sa			
		Ethyl acetate		3.0 Bs, 4.0 Mk and 4.0 Sa	Esc, Pv and Kla		
	Bulbs	Chloroform	0.05	0.63 Bs, Sa and Se	0.63 Spy and Pm	0.31 Tm, 0.63 Ca and Ct	[16,42]
	Leaves	Hexane	0.05	0.63 Bs and Sa	0.63 Spy, Pv, Pa and Pm		
	Tuber	Ethanol	0.0001–0.01	10 Sa	1 Esc, 1 Pa and 10 Kla		
		Ethyl acetate		5 Sa	7 Esc, 1 Pa and 1 Kla		
		n-Butanol		5 Sa	3 Esc, 3 Pa and 5 Kla		
	Leaves	Aqueous solvent	0.05	Bs, Mk and Sa	Esc, Pv and Kla	1 Ca	[6,43]
*Bulbine narcissifolia* (L.) Salm-Dyck		Methanol		6.0 Bs, 3.0 Mk and 4.0 Sa	Esc, Pv and Kla		
	Roots	Methanol	0.032	1.0 Bs, 3.0 Mk and 1.0 Sa	0.04 Ng and >8 Ou		
	Rhizomes	Methanol		2.0 Bs and 3.0 Mks	Pv and Kla		

Bacteria: Ab—*Actinomycetes brasielensis*; Acb—*Acinetobacter baumanii*; Bs—Bacillus subtilus; Cm—*Clavibacter michiganense*; Esc—*Escherichia coli*; Kla—*Klebsiella aerogenes*; Klp—*Klebsiella pneumoniae*; Ng—*Neisseria gonorrhoeae*; MRSA—methicillin-resistant; *Staphylococcus aureus*; Mk—*Micrococcus kristinae*; Ou—*Oligella ureolytica*; Sa—*Staphylococcus aureus*; Se—*Staphylococcus epidermidis*; Sp—*Streptococcus pneumoniae;* Spy—*Streptococcus pyogenes;* Pa—*Pseudomonas aeruginosa*; Ss—Shigella sonnei; Pm—Proteus mirabilis; Ec—*Erwinia carotovora*; Xc—*Xanthomonas campestris*; Pv—*Proteus vulgaris*. Fungi: Ct—*Candida tropicalis*; Tm—*Trichophyton mentagrophytes*; Tr—*Trichophyton rubrum*; Ca—*Candida albicans*. MIC = Minimum inhibitory concentration; (-): No activity; Values < 1 mg/mL are considered very active.

## Supplementary Materials

The following supporting information can be downloaded at https://www.mdpi.com/article/10.3390/plants14193045/s1. Figure S1: PRISMA flow diagram illustrating the search approach used to screen studies for eligibility in this review.
