# Supplementary material for: The Phytochemical Composition and Molecular Mechanisms Involved in the Wound Healing Attributes of Bulbine Species—A Critical Review
Source: Plants (Basel). 2025 Oct 1;14(19):3045. doi: 10.3390/plants14193045 (PMC12526425; doi:10.3390/plants14193045)
Supplement: Supplementary file 1 [file plants-14-03045-s001.zip › plants-3675299-supplementary.pdf]

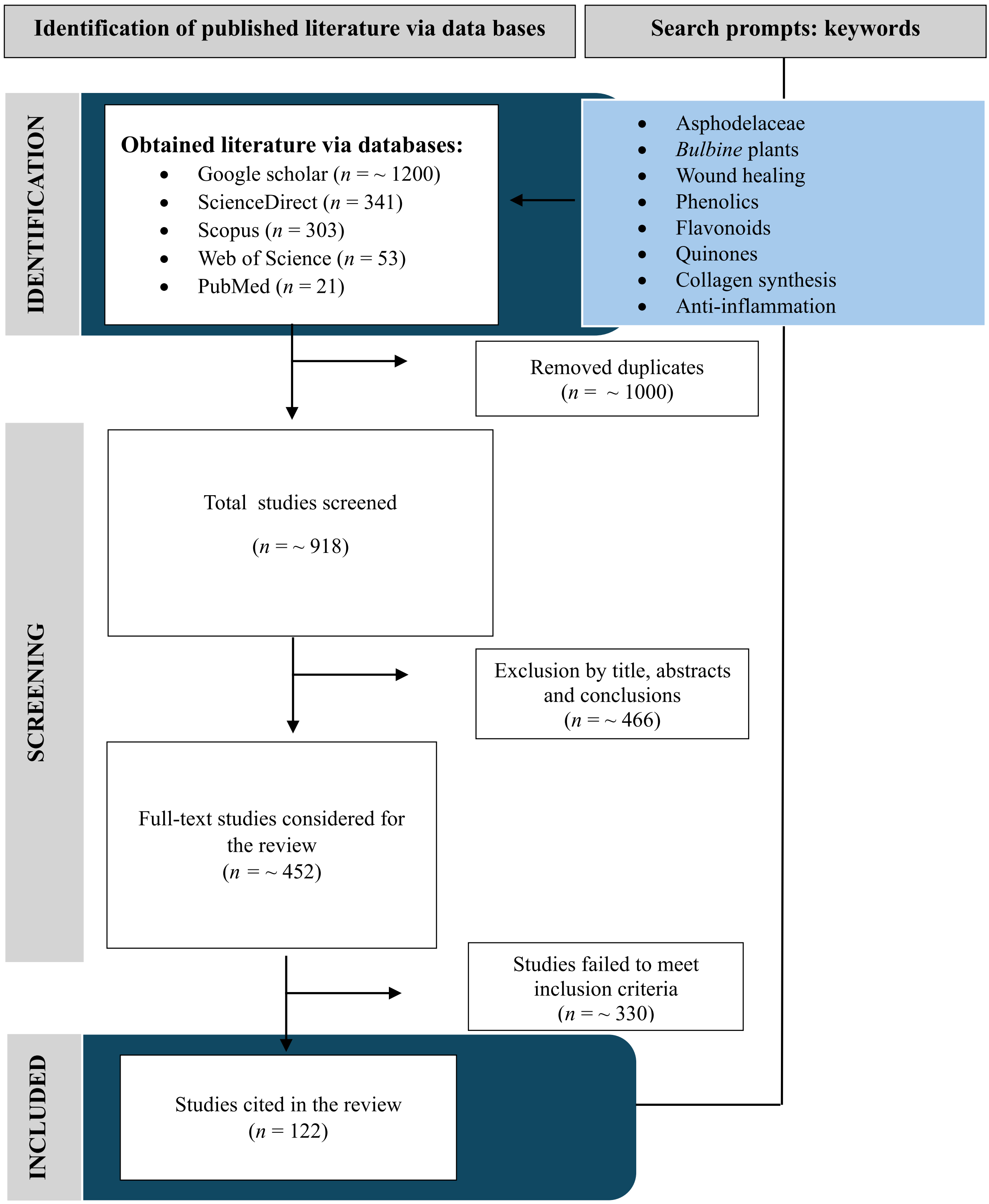

Supplementary Figure S1: PRISMA flow diagram illustrating search approach used to screen studies for eligibility in this review.
